# Herbivory mediates the response of below‐ground food webs to invasive grasses

**DOI:** 10.1111/1365-2656.70113

**Published:** 2025-08-29

**Authors:** Marco Fioratti Junod, Irene Cordero, Nadia Chinn, Jennifer Firn, Julia Holmes, Marcus Klein, Gabrielle Lebbink, Uffe N. Nielsen, Martin Schütz, Stephan Zimmermann, Anita C. Risch

**Affiliations:** ^1^ Community Ecology, Plant‐Animal Interactions Swiss Federal Institute for Forest, Snow and Landscape Research WSL Birmensdorf Switzerland; ^2^ School of Biology & Environmental Science Queensland University of Technology St. Lucia Queensland Australia; ^3^ Hawkesbury Institute for the Environment Western Sydney University Richmond New South Wales Australia; ^4^ Regional Ecosystem and Landscape Mapping Northern Territory Government Palmerston Northern Territory Australia

**Keywords:** grasslands, herbivory, invasion, soil ecology

## Abstract

1. Below‐ground food webs in grasslands are affected by both above‐ground herbivory and invasive plant species. However, the combined effects of these factors on soil organisms and their interactions with plant communities remain poorly understood.
2. We investigated how the invasive African lovegrass (ALG) influenced below‐ground food webs in south‐eastern Australian grasslands under different herbivory regimes. Using experimental exclosures, we established four treatments varying in herbivore presence (all animals present, non‐native mammals excluded, all mammals excluded, all above‐ground dwelling animals excluded) across sites dominated either by native kangaroo grass (KG; native sites) or co‐dominated by KG and ALG (invaded sites).
3. After 4 years, our results revealed that invasive grasses significantly altered the abundance and structure of soil bacteria, fungi, nematodes, arthropods and earthworms. These effects intensified along the gradient of herbivore exclusion. In parallel with herbivore exclusion, accumulated plant litter shifted decomposition from bacterial to fungal‐dominated, driven by changes in soil temperature, moisture and substrate, ultimately reshaping the soil biota assemblages.
4. Herbivory, particularly by both native and non‐native mammals, mitigated many of the adverse impacts of grass species invasion, with native and non‐native mammals acting additively. These findings underscore the intricate interplay between invasive grasses and herbivory, emphasising the importance of integrated management strategies to maintain the ecological balance of grassland ecosystems.

## Graphical abstract

This study investigates for the first time experimentally and in the field the interplay between plant invasion and herbivores of different guilds, shedding light on a complex network of below‐ground interactions and providing precious insights to land managers.

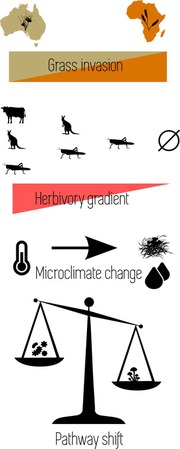

## 1 INTRODUCTION

Understanding ecosystem changes above‐ground requires insight into the role of soil organisms and their food webs in driving biological and chemical processes. These processes significantly impact ecosystem services and are crucial for conservation and management (Gundale & Kardol, 2021; van der Putten et al., 2016). In global grassland ecosystems, herbivory and invasive plant species—alongside fire—are major drivers of environmental change. While their combined influence on above‐ground biotic communities is well documented (Gornish & Ambrozio dos Santos, 2016; McGranahan et al., 2012), their impact on below‐ground communities remains poorly understood.

Above‐ground herbivory affects soil biota via root exudates and litter inputs (Bardgett, 2018), and generally increases springtail and earthworm abundances (Ashworth, Allen, et al., 2017; Dombos, 2001), bacterial diversity and microbial activity (Cheng et al., 2016; Patra et al., 2005). Conversely, herbivory tends to reduce mite and nematode abundances (Chachaj & Seniczak, 2005; Kinnear & Tongway, 2004; Zhou et al., 2023) and soil enzyme activity related to carbon (C) cycling (Hewins et al., 2015; Prieto et al., 2011; Xu et al., 2023). Soil detritivores and generalist predators mediate herbivory's top‐down effects on soil nutrient mineralisation and bottom‐up effects by influencing plant nutrient composition (Scheu, 2001; Whiles & Charlton, 2006). Moreover, the exclusion of vertebrate herbivores weakens the coupling between abiotic factors, vegetation and below‐ground biotic communities (Risch et al., 2018).

Invasive plants threaten ecosystem functions, biodiversity and nutrient availability (Teixeira et al., 2020; Vitousek et al., 1997). In grasslands, they can alter fire regimes (Brooks et al., 2004) and disrupt entire food webs (Foster et al., 2021). Invasive plants typically reduce soil mesofaunal abundance and bacterial diversity (Belnap et al., 2005; Gibbons et al., 2017), although some grasses enhance microbial enzyme activity and nutrient cycling (Zhou & Staver, 2019). However, their effects on soil communities vary by plant species (Porazinska et al., 2014).

In south‐eastern New South Wales, the invasive African lovegrass (*Eragrostis curvula*, ALG from now on) is encroaching on native Kangaroo grass (*Themeda triandra*, KG from now on), altering plant community composition (Schlierenzauer et al., 2021). Management strategies, such as spot spraying and increasing tree canopy cover, showed promise to reduce ALG, whereas slashing may encourage ALG growth (Firn et al., 2018). Grazing is another potential control method; though its effectiveness was conclusively recorded only when coupled with low‐rate fertiliser application (Firn & Buckley, 2010).

Australia's unique faunal history—lacking native ungulates and large herbivores since early human settlement (Roberts et al., 2001)—complicates the use of grazing for conservation. While livestock grazing has caused environmental damage, it is also recognised as a tool for managing grazing‐sensitive plant invasions (Lunt et al., 2007). Increasing herbivore diversity can enhance plant biodiversity by preventing single‐species grass dominance (Liu et al., 2015) and may compensate for the loss of medium‐sized mammals (Silcock et al., 2013).

However, herbivory effects depend on plant species palatability, grazing intensity and the functional role an animal plays within the system, yielding variable conservation outcomes (Dorrough et al., 2004; Koerner et al., 2018). Mammalian herbivory impacts are more pronounced in productive environments (Bakker et al., 2006; Bardgett & Wardle, 2003) with large species influencing vegetation through trampling and nutrient cycling (Meyer & Leroux, 2024), and smaller species through burrowing and foraging (Pascual et al., 2017). Their impacts can range from driving vegetation shifts (Brown & Heske, 1990) to having minimal effects (Burkepile et al., 2017). Finally, invertebrate herbivory, often overlooked, can significantly shape plant community dynamics (Risch et al., 2018) as insects have been shown to reduce plant biomass, alter plant species dominance (Coupe & Cahill, 2003; Ibanez et al., 2013) and therefore influence plant–soil feedbacks (Heinze & Joshi, 2018). Given global insect declines (Dirzo et al., 2014; Eisenhauer et al., 2019; Sánchez‐Bayo & Wyckhuys, 2019), understanding their role in ecosystem processes warrants further attention.

This study examined below‐ground trophic networks in a 4‐year herbivore exclusion experiment in KG‐dominated (‘native’) and KG‐ALG co‐dominated (‘invaded’) grassy woodlands of the Bega Valley, New South Wales (NSW), Australia. We assessed how herbivory and invasive grasses shape below‐ground arthropod, earthworm, nematode, fungal and bacterial communities. We hypothesised that invasion reduces bacterial diversity and soil arthropod abundance (Belnap & Phillips, 2001; Gibbons et al., 2017). We also predicted that herbivory will shift soil invertebrate composition, favouring springtails and earthworms while reducing mites and nematodes, alongside increasing bacterial diversity and metabolic activity (Ashworth, DeBruyn, et al., 2017; Chachaj & Seniczak, 2005; Dombos, 2001; Kinnear & Tongway, 2004; Zhou et al., 2023). In the paucity of sources dealing with the combined effects of herbivory and plant invasion on soil food webs, we hypothesise that positive feedback mechanisms are present that help the spread of invasive plants. Additionally, we investigated the mechanistic drivers of these changes, focusing on plant litter quantity as a key driver as litter composition has the potential to reshape soil webs (Cleveland et al., 2014) with higher litter inputs shifting community balance from mesodetritivores to macrodetritivores (Sauvadet et al., 2016). Furthermore, plant litter alters soil temperature and moisture, which in turn affects microbial and invertebrate communities (Borowik & Wyszkowska, 2016; Briones et al., 2009). Finally, we integrated our findings to identify shifts in fungal and bacterial degradation pathways under different herbivory treatments and grass invasion scenarios using a structural equation model approach (Wang et al., 2022; Zhang et al., 2019).

While the relation between groups of herbivores and plant invasion (Eldridge et al., 2020) or soil function (Eldridge et al., 2017) has been previously observationally investigated, to our awareness, this is the first study that experimentally assessed the interplay between functionally different non‐native and native animals and plant species invasion and includes the underlying mechanisms of changes observed in the field.

## 2 MATERIALS AND METHODS

### 2.1 Study sites

Herbivore exclosures were established in autumn 2019 on six active farms near Candelo, Bega Valley Shire, southeastern NSW, Australia (36°46′ S, 149°41′ E). These farms are located in lowland grassy woodlands dominated by C4 grasses, herbaceous plants and forest red gum (*Eucalyptus tereticornis*; Firn et al., 2018). On each farm, we selected two subsites in close proximity: one native and one invaded. Farms were subjected to extensive cattle and sheep (non‐native mammals) grazing, with mixed ALG control methods (roller wiping, slashing or no targeted measures) and unrestricted access to native (kangaroos, wallabies, wombats) and feral (non‐native) mammals (rabbits) and invertebrate herbivores. For an assessment of vegetation communities on the sites, see (Schlierenzauer et al., 2021).

### 2.2 Experimental design

**FIGURE 1:**
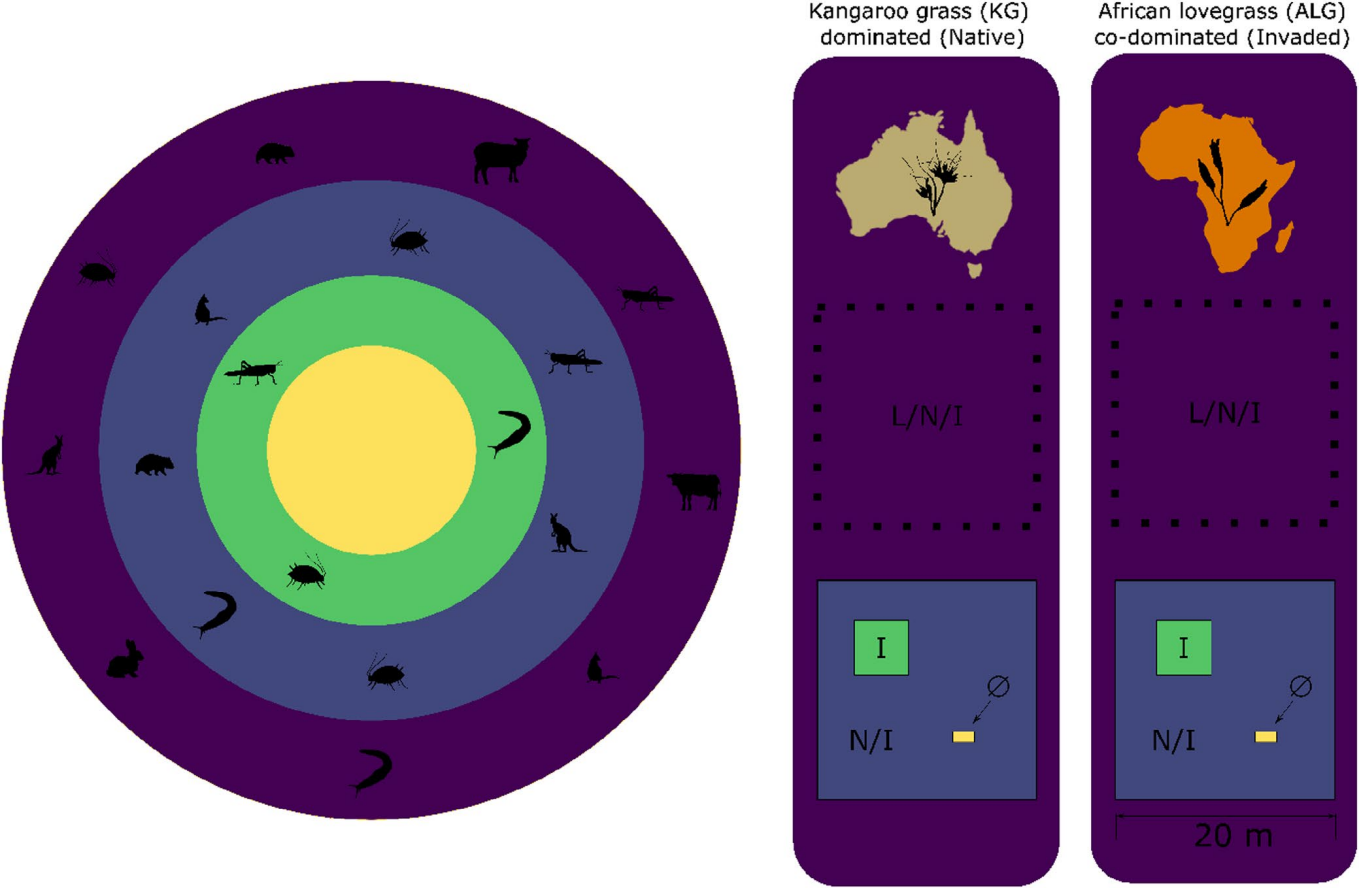
Schematic representation of the experimental layout. The experiment was replicated on six farms, and in each farm two subsites, were selected, one dominated by native kangaroo grass (KG) and one co‐dominated by invasive African love grass and native KG. On each farm a four‐tiered exclosure system was put in place, with access regulated by fences. In the outer, open area (‘L/N/I’), all animals could feed. Within the first fence, non‐native livestock and rabbits are excluded, but native large mammals still had access (‘N/I’). Within this fence, two further exclosures were erected, one excluding native mammal herbivory while open to invertebrates (‘I’), and the other barring access to all above‐ground feeding animals, which also included invertebrates (Ø).

A nested exclosure system (Figure 1) to experimentally exclude the functionally different herbivore guilds was established within each subsite:

L/N/I (20 × 20 m): unfenced area accessible to livestock and rabbits (non‐native mammals; L), native mammals (N) and invertebrates (I), located 5 m away from the main exclosure.

N/I (20 × 20 m): excluded L but allowed access to N and I. Fencing included wooden posts, wire without barbs at 20, 40, 80 and 130 cm height and a 20 mm mesh covering up to 40 cm above‐ground and extending belowground at a 20° angle to prevent access to burrowing rabbits.

I (5 × 5 m): nested within N/I, allowed access to I only. Fencing included wooden posts and wire mesh (5 × 20 cm mesh width) extending to a height of 150 cm to exclude wombats and wallabies by physical impediment and of kangaroos due to its small area.

∅ (1 × 2 × 1.5 m): also within N/I consisting of a wooden box covered on all sides with mosquito mesh, and treated bimonthly with permethrin to exclude soil‐hatched invertebrates. A second box with a 15 cm wide opening at the bottom of the long side of the box facing away from the prevailing rain and wind was also established to control for confounding effects of changes in microclimatic ∅ invertebrate exclusion interaction caused by the construction of the exclosure. No significant differences in moisture (*p* = 0.64) and above‐ground temperature (*p* = 0.80), as recorded by sensors, were detected between ∅ and the control box. Biomass comparison between the two is provided in Table S3.

### 2.3 Soil sampling

In February 2023, that is in the fourth year of excluding animals, four topsoil samples (5 cm diameter × 10 cm depth; soil corer AMS, American Falls ID, USA) were collected from each exclosure and composited by manually mixing. An aliquot was preserved in a sterile tube and immediately frozen for subsequent DNA extraction. The rest of the sample was kept for determining soil chemical properties. One undisturbed soil core was taken for bulk density and soil texture determination. An additional four cores (5 × 10 cm) were collected, composited and stored refrigerated at 4°C for nematode extraction and enzyme activity assays.

### 2.4 Soil physical and chemical analyses and soil enzymatic activities

The bulk density and soil water content were determined on the undisturbed cores after drying at 105°C to constant weight. The density of the fine earth fraction (<2 mm) was calculated after passing the samples through a 2‐mm mesh. Soil texture (percentage of sand, silt and clay) was determined using the sedimentation method according to Gee and Bauder (1986). The organic matter (OM) content of the soil was determined gravimetrically in a subsample dried at 105°C by combusting the sample at 400°C in a muffle furnace for 16 h (Nelson & Sommers, 1996).

For soil chemical analysis, the samples were dried at 60°C to constant weight and passed through a 2 mm sieve. The pH was measured in a 1:1 soil slurry in 0.01 M CaCl_2_ (Kissel et al., 2009). Subsamples were ground to a fine powder (<50 μm) in a ball mill (Retsch MM 400, Düsseldorf Germany) to determine total C and N concentrations (Carlo Erba Instruments NC 2500, Milan, Italy). Soil organic C concentration was measured with the same device after fumigating overnight a subsample with concentrated HCl in a desiccator (Walthert et al., 2010).

Total organic P was determined by the ignition method (Saunders & Williams, 1955) as follows: organic P was converted to inorganic P by high temperature oxidation; organic P was then determined by the difference between the amounts of H_2_SO_4_‐extractable P for the ignited (total P) and unignited (inorganic P) soils (Kuo, 1996). These fractions were determined colorimetrically in the extracts with the Malachite Green reagent. Available P was extracted with 0.5 M sodium hydrogen carbonate at pH 8.0 (Kuo, 1996) and measured spectrophotometrically by the molybdate‐ascorbic acid method (Murphy & Riley, 1962) using a Cary 60 UV–vis spectrophotometer (Agilent Technologies, Santa Clara, US).

Potential microbial enzyme activities were determined with microplate colorimetric and fluorometric methods. Fluorescent‐tagged substrate analogues were used for alkaline phosphatase (MUB‐phosphate), β‐glucosidase (4‐MUB‐β‐D‐glucopyranoside), β‐xylosidase (4‐MUB‐β‐D‐xylopyranoide), N‐acetylglucosaminidase (4‐MUB‐N‐acetyl‐β‐D‐glucosaminide), leucine aminopeptidase (L‐leucine‐AMC) and a colour‐forming substrate was used for phenol oxidase (3,4‐dihydroxyphenylalanine [L‐DOPA]) and phenol peroxidase (L‐DOPA with H_2_O_2_). A slurry obtained from mixing 10 g of fresh soil with 300 mL of acetate buffer at pH 5.0 was loaded in triplicate 200 μL aliquots in the microplate wells and incubated with 50 μL of each substrate analogue at constant shaking for 90 min at 20°C. Ten microlitres of 0.5 N NaOH was added prior to measurement to increase pH and stop the reaction. Measurement of fluorescence and colour was carried out on a Tecan Infinite 200 plate reader (Männedorf, Switzerland) according to the protocol laid out by (Frossard et al., 2012).

### 2.5 Below‐ground arthropod sampling

Below‐ground pitfall traps with sampling ports extending 2.5 cm in width and 0–10 cm in the soil profile (Fioratti Junod et al., 2023) were deployed in February 2023 in each of the treatments in each subsite and left in place for 1 week. The collecting tubes, filled with ethanol, were recovered and their contents were transferred to a Petri dish. All specimens were examined under a stereomicroscope with contrasting backgrounds and identified to family level based on morphological characters using a variety of resources and dichotomous keys (Bellinger et al., 2024; Hopkin, 2007; Shepherd & Crotty, 2018).

### 2.6 Earthworm sampling

A soil monolith of 20 × 20 × 20 cm was extracted in February 2023 at a randomly selected location within each treatment plot at each subsite, with the exception of the ∅ exclosures due to the limited dimensions. The resulting hole was checked for permanent anecic earthworm burrowing tunnels. The clod was disaggregated on a contrasting surface. All earthworms were sorted manually, stored in plastic tubes and identified within 24 h (Baker & Barrett, 1995).

### 2.7 Nematode extraction and count

A total of 225 g of fresh refrigerated soil were used for nematode extraction with Baermann funnels in three batches of 75 g each. The soil samples were kept for 4 days in the funnels and watered twice a day. The first two extractions were combined and used for counting nematodes on a gridded Petri dish under a stereomicroscope at 40× magnification. The third batch was concentrated to a volume of 2 mL for downstream molecular analyses (see below). A soil aliquot of 10 g was used to determine soil moisture content.

### 2.8 DNA extraction and bioinformatics

DNA for fungal and bacterial profiling was extracted from a 0.25 g subaliquot of frozen soil with the DNeasy PowerSoil Pro kit (Qiagen, Hilden, Germany) according to the manufacturer's instructions. The same kit was used to extract DNA from bulk nematode samples obtained with Baermann funnels, after concentration of 2 mL aliquots to 250 μL following centrifugation at 5.500 g for 5 min and removal of the supernatant. The DNA yield was quantified with a Qubit™ dsDNA High Sensitivity kit and instrument (ThermoFisher, Waltham, USA). DNA extracts were then sent to Novogene Ltd. (UK) for amplification and Illumina‐based sequencing on a paired‐end 250‐bp platform.

For bacteria, a fragment covering the V3 and V4 regions of the 16s rRNA gene was amplified by polymerase chain reaction (PCR) using the primer pair 341 F/806 R (CCTAYGGGRBGCASCAG/GGACTACNNGGGTATCTAAT), modified from Herlemann et al. (2011). For fungi, a section of the internal transcribed spacer 2 (ITS2) DNA was amplified with the primers ITS3‐2024F (GCATCGATGAAGAACGCAGC) and ITS4‐2409R (TCCTCCGCTTATTGATATGC), modified from White et al. (1990). For nematodes, the primers NemF (GGGGAAGTATGGTTGCAAA) and 18Sr2b (TACAAAGGGCAGGGACGTAAT) were used for amplification of the v7–v8 region of the 18S rRNA gene (Sapkota & Nicolaisen, 2015).

The 16s and ITS2 sequences were filtered and denoised according to the DADA2 pipeline (Callahan et al., 2016), which allowed us to retain, respectively, 82% and 87%, resulting in 34,761 and 14,412 ASVs. Taxonomic assignment was performed against the SILVA (version 138.2) and Unite (version 10.0) databases. For bacteria, inferred EC functional profiles were extracted with the PICRUSt2 pipeline (Douglas et al., 2018, 2020; Supporting Information S1). For fungi, guild and trophic mode information was extracted with the FUNGuildR package in R (version 0.2.0.9; Nguyen et al., 2016). Nematode sequence analysis was performed only with the reverse reads, due to non‐overlapping reads and low quality of forward reads. Sequences were analysed using the DADA2 pipeline (Callahan et al., 2016) with default parameters. After filtering and de‐noising steps, 64% of the reads were retained, and 3567 ASVs were identified. Taxonomic identification was performed by the IDTAXA taxonomic classification method in DECIPHER (Wright, 2016) package using the SILVA database.

### 2.9 Microclimate monitoring and plant biomass sampling

Each treatment plot had a T‐MS4 data logger (Tomst, Czech Republic) deployed, recording soil temperature (0–10 cm depth) and moisture (0–10 cm) every 15 min throughout the experiment. In May 2023, two 20 × 100 cm strips per plot were harvested. Vegetation was sorted into KG, ALG, other graminoids, forbs and litter, dried (60°C, 48 h) and weighed. The values for the two strips were averaged and extrapolated to square metre.

### 2.10 Statistical analysis

The effects of treatment on continuous variables were tested by fitting mixed effects linear models having herbivory treatment (categorical variable with four factors) and ALG invasion (binary) as fixed explanatory variables and farm as a random factor. The analysis was performed with the lme4 package (version 1.1‐35.3; Bates et al., 2014). For discrete variables, an underlying Poisson distribution was assumed. Extraction of effects and marginal means was performed with the packages lmerTest (version 3.1.3; Kuznetsova et al., 2017) and emmeans (version 1.10.2; Lenth, 2022). Taxa associated with specific treatments or with invasion were identified with the same models, with Bonferroni correction to account for multiple comparisons.

Rarefaction‐corrected Shannon's diversity indices and species richness were calculated with Hill's numbers with the R package hillR (version 0.5.2; Li, 2018) and Functional diversity with the pd function of the R library (version 1.8.2; Kembel et al., 2010) on a clustered UPGMA dendrogram generated on a Gower's dissimilarity matrix of the functional trait tables.

Candidate PICRUSt2 pathways for bacteria clearing the 0.001 statistical threshold for the given explanatory variable were identified as differentially expressed; for the herbivory treatment, only the pathways showing the same effect direction for the three exclosure treatments compared with the L/N/I treatment were taken into account.

PERMANOVA (Permutational analysis of variance), using farms as strata, canonical correspondence analysis and environmental vector fitting on community data were performed, respectively, with the functions adonis2, cca and envfit of the R package vegan (version 2.6.4; Oksanen, 2018; Oksanen et al., 2008).

Structural equation modelling was performed with the function fit from the R package lavaan (version 0.6.17; Rosseel, 2012). A selection of variables measured across all samples and their interactions was made according to existing literature (Table S1). The variables were fit into a regression network after standardisation.

A one‐knot splined linear model was fitted to the data for soil temperature in the year preceding the sampling, measured at 10 cm beneath the surface, with a knot at the seasonal variation breaking point on 27 July 2022, and farm, invasion and herbivory exclusion as explanatory variables. For every day, a single input value was obtained by averaging all temperature values collected at 15‐min intervals throughout the day. A similar approach was used to model soil moisture values in the 0–10 cm interval, but the more complex general rainfall pattern required two additional knots, set at 15 October and 24 December 2022.

All analyses, as well as data cleaning and processing, were performed with R version 4.3.2 (R Core Team, 2021) in an RStudio Chocolate Cosmos release (Posit team, 2024) environment.

### 2.11 Ethical approval and authorisations

No ethics approval was required for this research. Written agreements were reached with all involved land owners for the placement of the exclosures on their farms.

## 3 RESULTS

### 3.1 Soil microclimate conditions

**FIGURE 2:**
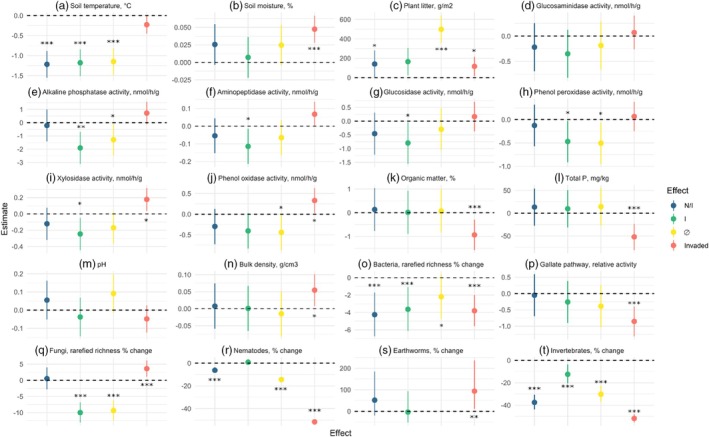
Modelled size effects and confidence intervals for selected biotic and abiotic parameters. (a) Soil temperature at 10 cm depth. (b) Average topsoil moisture. (c) Dry litter biomass. (d) β‐1,4‐N‐acetylglucosaminidase activity. (e) Alkaline phosphatase activity. (f) Leucine aminopeptidase activity. (g) Glucosidase activity. (h) Phenol peroxydase activity. (i) Xylosidase activity. (j) Phenol oxidase activity. (k) Organic matter (LOI). (l) Total soil phosphorus. (m) Soil pH. (n) Soil bulk density. (o) Bacterial ASVs, rarefied richness. (p) Gallate degradation pathway I. (q) Fungal ASVs rarefied richness. (r) Soil nematodes per kg off dry soil. (s) Earthworms, individuals per square metre. (t) Invertebrates recovered per pitfall trap. In blue N/I, in green I, in yellow ∅, in red Invaded. Asterisks refer to significant difference compared with the control (L/N/I, Native).

Soil temperature (10 cm depth) was lower in herbivory treatment plots. The N/I treatment had 1.22°C (*p* < 0.001), the I plots 1.18°C (*p* < 0.001) and the Ø plots 1.15°C (*p* < 0.001) lower temperature than the L/N/I plots (Figure 2a; Figure S1a). Additionally, invasion was associated with a cooling of 0.23°C (*p* < 0.05) compared with native plots. Invasion also led to an increase of 4.8% (*p* < 0.001) soil moisture compared with native vegetation (Figure 2b; Figure S1b). The effect of herbivory treatments on soil moisture was not significant, with a general increase in exclosures compared with the open plots.

### 3.2 Plant litter biomass

Invasion resulted in an increase of 116.2 g m^−2^ of plant litter (*p* < 0.05) in the invaded compared with the native plots, whereas the herbivory exclosures treatments led to increases in litter biomass of 172.45 g m^−2^ for the N/I treatment (*p* < 0.05) and 498.56 g m^−2^ for the Ø treatment (*p* < 0.001) compared with the L/N/I plots (Figure 2c).

### 3.3 Potential microbial enzyme activities

Glucosaminidase activity did not show any significant change. For alkaline phosphatase, leucine aminopeptidase, glucosidase and phenol peroxidase, only herbivore exclusion (not invasion) was a significant predictor (Figure 2e–h), resulting in significantly reduced activity in the I and Ø exclosures for phenol peroxidase (*p* < 0.05 and *p* < 0.05) and alkaline phosphatase (*p* < 0.01 and *p* < 0.05), and in the I treatment only for glucosidase (*p* < 0.05) and leucine aminopeptidase (*p* < 0.05) compared with the L/N/I plots. For xylosidase and phenol oxidase, both herbivore exclusion and grass invasion were significant (Figure 2i,j). Invasion was associated with substantial activity increases in both enzymes (*p* < 0.05 and *p* < 0.05) compared with native plots, while the I treatment led to significantly reduced activity in xylosidase (*p* < 0.05) and the Ø treatment in phenol oxidase (*p* < 0.05) compared with the L/N/I plots.

### 3.4 Physical and chemical soil properties

Soil OM was not significantly affected by herbivore exclusion, but was 0.94% lower in the invaded than in the native plots (*p* < 0.01; Figure 2k). The same trend was observed for total P, with 51.91 mg kg^−1^ lower values in invaded than in native plots (*p* < 0.001; Figure 2l). No significant differences were found between herbivore treatments and plant invasion for pH (Figure 2m).

The bulk density of topsoil did not show any effect of herbivory exclusion either, but increased by 0.06 g cm^−3^ in invaded (*p* < 0.05) compared with native plots (Figure 2n).

### 3.5 Bacteria

**FIGURE 3:**
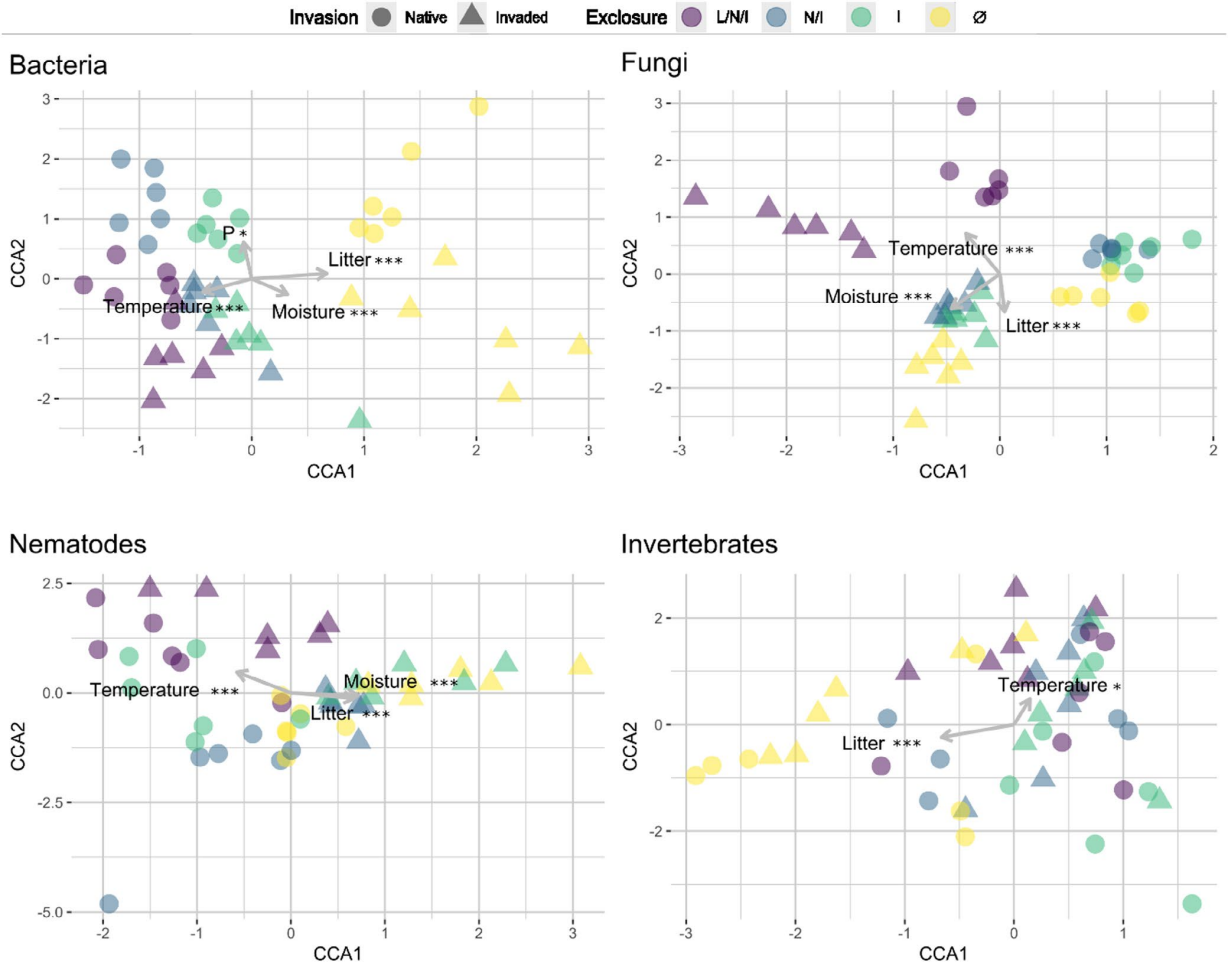
Constrained coordinate analysis of bacterial, fungal, nematode and invertebrate communities. The ordination was constrained to the experimental variables (herbivory exclusion and *African lovegrass* invasion). The environmental variables showing significant regression coefficients with the linear combination scores are shown as projected vectors. Asterisks indicate the significance of the associated parameter (*p < 0.05, **p < 0.01, ***p < 0.001).

PERMANOVA shows that both herbivory exclusion (*p* < 0.001) and invasion (*p* < 0.01) were significant factors in shaping the bacterial assemblage, respectively. Plant litter biomass (*r* ^2^ = 0.51; *p* < 0.001), average soil temperature (*r* ^2^ = 0.22, *p* < 0.01), total P (*r* ^2^ = 0.12, *p* < 0.05) and moisture (*r* ^2^ = 0.36, *p* < 0.001) were significant predictors of structural changes in bacterial community composition (Figure 3a).

Invaded plots had lower rarefied numbers of ASVs (*p* < 0.001; Figure 2n), just as the ones where herbivores were excluded (*p* < 0.001; *p* < 0.05 for Ø). *Gemmatimonadota* were less abundant under invasive grasses (*p* < 0.001) and *Methylomirabilota* became progressively more abundant with herbivory exclusion (*p* < 0.01 for the I treatment; *p* < 0.001 for the Ø exclosures) (Figure S2).

As for inferred functional profiles (Supporting Information S1), the superpathway of fucose and rhamnose degradation and the mycolyl‐arabinogalactan‐peptidoglycan complex biosynthesis were found to be less expressed in herbivore exclosures; whereas the opposite was observed for dTDP‐β‐L‐rhamnose biosynthesis, L‐isoleucine biosynthesis IV, guanosine diphosphate mannose biosynthesis, chlorosalicylate degradation and chorismate biosynthesis from 3‐dehydroquinate. Gallate degradation (Figure 2p), methylgallate degradation, mycolyl‐arabinogalactan‐peptidoglycan complex biosynthesis and plant‐derived steroid degradation pathways were all found to be suppressed under invasion.

A PERMANOVA approach applied to the general enzyme expression matrix revealed that herbivory exclusion (*p* < 0.01) but not invasion was a predictor of bacterial community functional variability. Conversely, Faith's PD of the bacterial functional profile showed a near‐significant effect in invaded plots (*p* < 0.1) but no effect of herbivore exclusion (Figure S4).

### 3.6 Fungi

Invasion (*p* < 0.001) and—marginally—herbivory exclusion (*p* < 0.1) were contributors to fungal assemblage formation. Again, plant litter biomass (*p* < 0.001, *r* ^2^ = 0.45), average soil temperature (*p* < 0.001, *r* ^2^ = 0.71) and moisture (*p* < 0.001, *r* ^2^ = 0.57) explained these community differences (Figure 3b). A reduced number of fungal ASVs was recorded under the I and Ø treatments (*p* < 0.001), whereas invasion increased the richness (*p* < 0.001). *Chytridiomycota* was the only clade whose relative abundance was significantly enhanced in invaded compared with native plots (*p* < 0.01; Figure S4a).

All levels of herbivory exclusion resulted in declines in guild Faith's PD diversity, although only for the I exclosure was the effect significant (*p* < 0.05). The relative read abundance of saprotrophs was enhanced in invaded plots by 5.2% (*p* < 0.05; Figure S4b).

### 3.7 Nematodes

Soil nematode abundance was generally negatively affected by invasion, with an estimated loss of 3046 individuals per kg of dry soil (*p* < 0.001) compared with native plots. Soil nematode abundance was also negatively affected by the exclosure treatments, with significant decreases brought about by the N/I (−270 individuals, *p* < 0.001) and Ø (−623 individuals, *p* < 0.001) exclosures (Figure 2r) compared with the L/N/I plots. As for nematode community structure, both invasion (*p* < 0.001) and herbivory exclusion (*p* < 0.001) were predictors of nematode composition, capable of explaining 6.8% and 12.5% of the total variability, respectively. Once more, plant biomass litter (*r* ^2^ = 0.47, *p* < 0.001), average soil temperature (*r* ^2^ = 0.51, *p* < 0.001) and moisture (*r* ^2^ = 0.55, *p* < −0.001) emerged as significant predictors of changes in nematode community composition (Figure 3c).

Rarefied nematode species richness was found to be positively associated with invasion (7.25 additional nematode species, *p* < 0.05; Table S1) and negatively with herbivore exclusion, namely the I (−11.9 species, *p* < 0.05) and Ø (−12.9 species, *p* < 0.01) treatments. No significant predictor of Shannon's diversity was identified in the experimental variables. Among nematode orders (Figure S5), the relative abundance of *Rhabtidida* was higher under invasion (+13%, *p* < 0.01) as well as herbivory exclusion, with a significant effect measured in the N/I treatment (+13%, *p* < 0.05) compared with the L/N/I plots.

### 3.8 Earthworms and soil arthropods

All recovered specimens were non‐native lumbricids, with all the adults identified as *Aporrectodea* sp. Earthworm numbers were higher in invaded (+16.2 individuals per square metre, *p* = <0.01; Figure 2s) than in native plots. No significant effects were associated with herbivory exclusion. Below‐ground arthropod abundance was significantly and negatively affected by invasion (−67.5 specimens per trap, *p* < 0.001; Figure 2t) as well as by herbivory exclusion, with a loss of 42.4 specimens for the N/I treatment (*p* < 0.001), −13.7 for the I treatment (*p* < 0.001) and −34.1 for the Ø treatment (*p* < 0.001). The rarefied species richness and Shannon's diversity index were not found to be predicted by grass invasion or herbivore exclusion suppression.

The community composition of soil arthropods was shaped by invasion (5.0%, *p* < 0.01) and herbivory exclusion (12.7%, *p* < 0.001). Plant litter biomass (*p* < 0.001, *r* ^2^ = 0.47) and average soil temperature (*p* < 0.05, *r* ^2^ = 0.25) explained these differences (Figure 3d).

We detected a strong effect of the exclusion treatment and invasion on the springtail family Entomobryidae (Figure S6). Invasion (*p* < 0.001, −8.86 individuals), N/I exclosure (*p* < 0.001, −4.43) and I exclosure (*p* < 0.001, −7.94) led to significant declines in the number of Entomobryidae specimens, respectively; the Ø treatment, conversely, resulted in a substantially increased abundance of individuals in this group (28.2 more individuals, *p* < 0.001).

### 3.9 Pathway modelling

**FIGURE 4:**
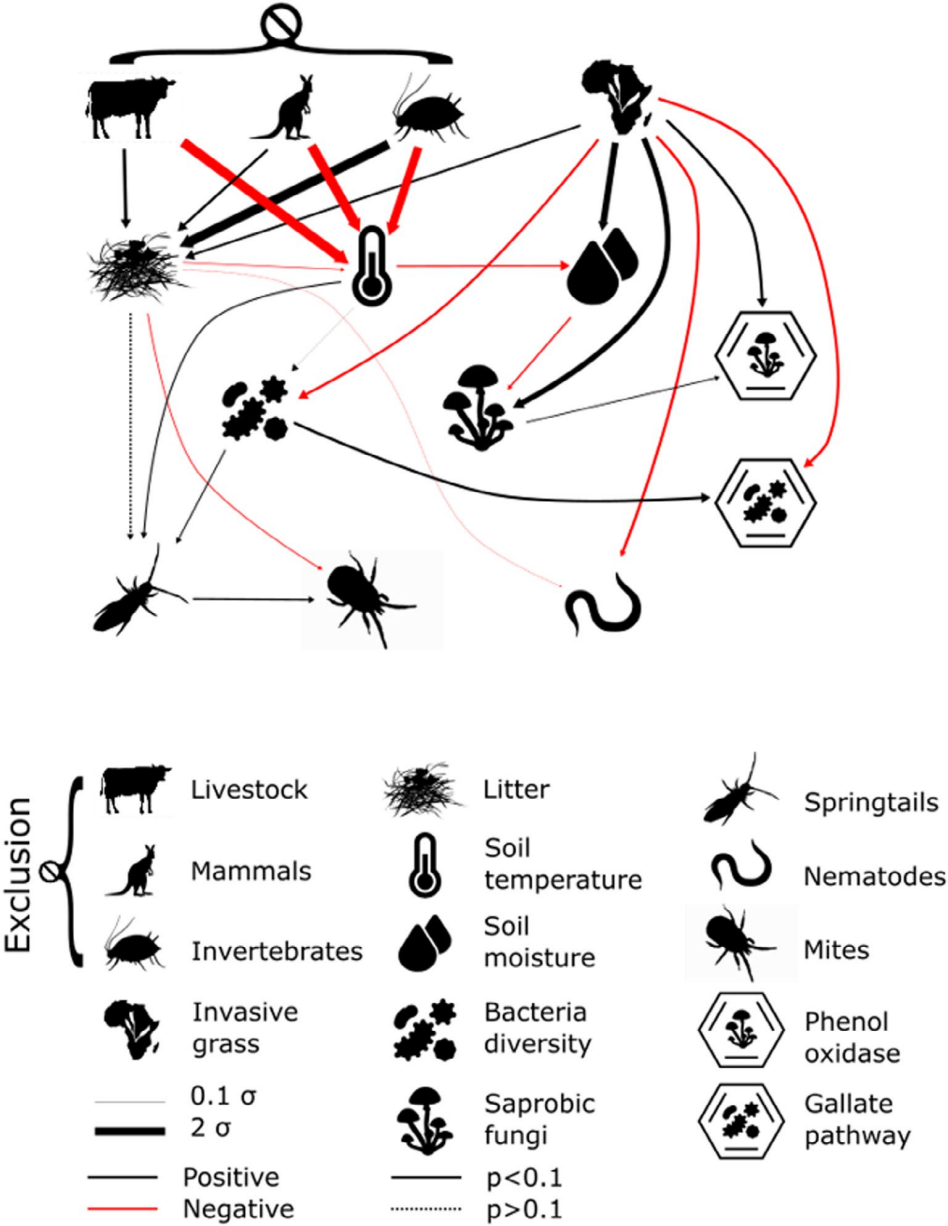
Conceptual representation of a structural equation model linking herbivore exclusion, grass invasion, soil microclimatic and soil biotic parameters. The sign of the correlation is indicated by line colour and the magnitude by line thickness. N/I, I, Ø exclosure treatments and grass invasion were defined as dummy variables. All other variables were standardised. The diagnostic parameters of the model are comparative fit index 0.998, root mean square error of approximation 0.012.

Herbivore exclusion was associated with an increase in accumulated plant litter biomass and a decrease in soil temperature (Figure 4). Plant litter biomass was, in turn, negatively associated with the abundance of nematodes and mites. Only part of the variability in soil moisture was explained by its negative relation with soil temperature, with a stronger positive association with invasion. A reduction in bacterial diversity and a parallel increase in the relative abundance of fungal saprotrophs was strongly related to invasion as opposed to litter biomass. This is consistent with different biochemical pathways enhanced or repressed by ALG presence, namely phenol oxidase and gallate metabolism. Our SEM resulted in a root mean square error of approximation of 0.012 and a comparative fit index of 0.998, indicating a good fit.

## 4 DISCUSSION

### 4.1 Impact of plant invasion on below‐ground food webs

The hypothesis of a general reduction in diversity, but not necessarily function, brought about by plant invasion in below‐ground communities was fully supported by our findings. The picture for the forecasts of herbivory exclusion is less univocal. While herbivore exclusion reduced springtail numbers, bacterial richness and—partly—enzyme activity, it did not bring about increases in nematode numbers or widespread reductions in bacterial Shannon's diversity. As for the interaction effects between herbivory and plant invasion, the existence of positive feedback mechanisms between invasive plants and soil communities is tentatively confirmed, in the light of the recruitment of invasive earthworms, but it seems to suffer disruption or attenuation in the presence of above‐ground herbivory.

The higher cover of ALG in invaded plots led to a decrease in soil total P, attributable to more vigorous P uptake, and a marked increase in plant litter biomass. Grass invasion‐driven plant litter accumulation had cascading effects on the soil and its biotic components. In addition to the physical effects of litter increase, like higher soil moisture and lower soil temperature, the contribution of invasive grass litter was likely acting through the channel of chemical variation in the substrate. The increase in xylosidase and phenol oxidase activity (Tian & Shi, 2014) together with an increase in fungal saprotrophs and a general restructuring of the fungal community driven by ALG invasion point to the different biochemical profile of the plant litter entering the system with invasion (Castellano et al., 2015; Firn et al., 2012; Masubelele & Bond, 2022). Such a take‐over of different fungal mutualists (Reinhart & Callaway, 2006) and the rapid disruption of existing fungal communities (Řezáčová et al., 2021) are commonly observed following plant invasion.

The observed bacterial community restructuring under ALG invasion detected in our study is also part of the same overarching pattern (Belnap et al., 2005; Cheng et al., 2016; Gibbons et al., 2017). A general decline in bacterial ASVs and changes in bacterial metabolic signals, like the decline in gallate pathway markers (Masubelele & Bond, 2022; Schmidt et al., 2013), also point in the direction of a marked shift of the brown food web the bacteria towards the fungal channel with ALG invasion. Repercussions of the invasion are visible further up the soil food web, with significant declines in nematode and arthropod abundance. As for nematodes, the effect of invasive plants on their number and community composition has yielded contrasting results in the literature and may be context and plant species dependent (Zhou et al., 2023; Zolda, 2006). The fact that nematode taxonomic richness was higher under ALG invasion indicates that a more heterogeneous feeding substrate might have favoured the recruitment of a more varied community, even in the context of a general reduction in abundance. Nevertheless, while high‐resolution data on taxonomic groups and feeding guilds is limited, given the higher relative abundance of bacterivores in nematode communities (van den Hoogen et al., 2020), the decrease in total nematode abundance could be interpreted as another indication of a shift from bacterial to fungal pathways associated with plant litter accumulation following ALG invasion. In addition, the detected decline in the epi‐edaphic and hemi‐edaphic collembolan group *Entomobryidae* in invaded plots was consistent with observations made for the clade in association with higher fungal/bacterial ratios (Maaroufi et al., 2018). Similar contractions have been shown in studies assessing impacts on soil fauna of other invasive grasses (Belnap & Phillips, 2001; De Almeida et al., 2022), with epi‐edaphic collembola being particularly affected and reacting rapidly to changes in plant community composition.

Finally, for earthworms, the association between an opportunistic invasive genus and the abundance of a feeding substrate, in this case ALG litter, was evident in our study and is well documented across gradients of degradation in south‐eastern Australian grasslands (Carnovale et al., 2015). While earthworms in general increase soil macropore space, the presence of invasive earthworm species has been shown to homogenise and compact the soil matrix and reduce large soil pores (Chauvel et al., 1999), which may be bottom‐up drivers of the higher bulk density that we recorded under ALG invasion. In general, invasive earthworms have the capacity to select for plant traits and favour in turn invasive plant species (Thouvenot et al., 2021). ALG may therefore benefit invasive earthworms with a potential positive feedback for the plant itself due to greater soil OM breakdown and nutrient availability.

### 4.2 Differential impact of functional groups of herbivores on below‐ground food webs

As observed for ALG invasion, we also found increased litter accumulation with herbivore exclusion. This emerged as a strong driver for shaping the bacterial community, with bacterial assemblages in the ∅ treatment, where we found the largest change in litter accumulation with exclusion, being particularly divergent. Most likely, the changes in available substrate (litter accumulation), together with reallocation of carbon to the rhizosphere, were the primary channel through which invertebrate herbivory affected below‐ground communities, as previously suggested by Wardle and Bardgett (2008). Unlike plant invasion, herbivore exclusion also significantly affected the functional profile of bacterial assemblages, indicating a gradual reorganisation of these communities in response to the exclusion of the different herbivore groups. We found that herbivory exclusion had a less discernible effect on the structure of the fungal community, which is in accordance with published literature: in the absence of grazing, there is only slow restructuring time of fungal communities (van der Heyde et al., 2017). Risch et al. (2018) also reported a high impact of invertebrate herbivore exclusion on plant biomass accumulation, but in their study conducted in the Swiss Alps, both the bacterial and fungal communities remained unchanged after 5 years of herbivore exclusion. This difference to our findings could be related to the much lower temperatures in the subalpine study in Switzerland than ours, conducted in eastern Australia.

As expected for the mesofauna in non‐peaty soils, we found a progressively lower abundance of soil arthropods related to increases in soil OM content with each additional level of herbivore exclusion (George et al., 2017). In the only other comparable study that assessed how mammalian and invertebrate herbivores affect soil arthropods, the largest difference in soil arthropod abundance was detected when all herbivores were excluded (Vandegehuchte et al., 2015), highlighting the dramatic direct and indirect impact this clade has on the availability of detritus (Zou et al., 2016). Conversely, the increased abundance of *Entomobryidae* in our ∅ plots compared with the other herbivory exclusion treatments could indicate the presence of untapped food sources due to the suppression of their above‐ground detritivore counterparts. A higher proportion of plant material normally consumed by Orthopterans or above‐ground dwelling Symphypleona springtails would have been made available.

Herbivore exclusion also negatively affected nematode abundances and species richness and was a strong driver of changes in nematode community composition. While most literature reported herbivory to be negatively associated with nematode abundance in soil (Zhou et al., 2023; Zolda, 2006), the phenomenon is likely context dependent. The reduction of resource availability (Andriuzzi & Wall, 2018) and depletion of microbial biomass with herbivore exclusion (Wang et al., 2006) may have driven the opposite trend observed in our study.

### 4.3 Joint effects of herbivore exclusion and plant invasion on below‐ground food webs

Literature covering the joint effects of herbivore exclusion and plant invasion on soil food webs is extremely limited (Gao et al., 2023; Zhou et al., 2017) and confined to single invertebrate herbivores in highly controlled indoor conditions. In these settings, positive feedbacks of herbivory on the fitness of invasive plants, mediated by soil communities, were registered. Our study, the first in situ experiment combining plant invasion and the exclusion of functionally different mammals and invertebrates, provided strong evidence that herbivory is effective at buffering the impact of ALG on the below‐ground food web. For the vast majority of examined parameters, the trend brought about separately by invasion and herbivory was directionally the same and suggested that the mere quantity of litter biomass was the mechanistic force behind chemical and biotic change. For the few parameters for which we found diverging changes (e.g. enzyme activities and earthworm abundance) or interactions (e.g. bacterial functional diversity) between the impact of plant invasion and herbivory, the likely explanation can be found in the different chemical composition of the substrate provided by ALG litter, which is higher in C:N than KLG (Masubelele & Bond, 2022). The magnitude of change induced by ALG litter gets amplified by the larger biomass brought about by grazing exclusion, compounding its effect.

Furthermore, it might be possible that different growth allocation and defence mechanisms (Endara & Coley, 2011) of KG and ALG were at the base of some observed patterns in microbial metabolic functions in our combined invasion and herbivore exclusion experiment. The metabolic signals were the endpoint of a whole‐trophic chain shift triggered by the compound effect of plant litter quantity and, speculatively, quality. Accumulation of ALG litter with increasing herbivory exclusion likely triggered a reduction in soil mesodetritivores and nematodes driven by a structural shift and diversity reduction in bacterial communities. The bacterial communities, in turn, were gradually replaced in their functions by fungal degraders and soil macrodetritivores. Unfortunately, we cannot compare these findings to the literature; we are the first to assess such a complex network of interactions simultaneously under plant invasion and herbivore exclusion.

### 4.4 Implications for conservation

Our findings highlight that herbivory can mitigate the negative effects of ALG invasion, with evidence suggesting that livestock grazing may complement the role of native wild mammals in maintaining soil biotic integrity and therefore ultimately soil health. Thus, our findings underscore the potential for livestock grazing as a conservation tool in landscapes impacted by invasive grasses, particularly in human‐modified ecosystems with a history of livestock grazing. While our findings stem from a case study, the ubiquity of ALG as a global invasive grass and of such environments means they have potential for much wider applicability. Future research should focus on elucidating the biochemical mechanisms underlying invasive grass‐driven trophic shifts and quantifying herbivory pressure on both native and invasive grasses. Identifying key invertebrate herbivores will further enhance our ability to manage invasive grass impacts effectively across diverse ecosystems.

## AUTHOR CONTRIBUTIONS

Jennifer Firn, Anita C. Risch and Martin Schütz conceptualised the project. Anita C. Risch, Jennifer Firn, Uffe N. Nielsen, Gabrielle Lebbink, Stephan Zimmermann and Marco Fioratti Junod defined the methodology. Anita C. Risch, Martin Schütz, Stephan Zimmermann, Gabrielle Lebbink, Nadia Chinn and Marco Fioratti Junod designed the sampling process and carried out data collection. Nadia Chinn, Gabrielle Lebbink and Jennifer Firn carried out the vegetation survey. Stephan Zimmermann, Julia Holmes, Marcus Klein, Uffe N. Nielsen, Irene Cordero and Marco Fioratti Junod performed the chemical, biological and molecular assays. Marco Fioratti Junod sorted and identified the invertebrates. Marco Fioratti Junod and Irene Cordero analysed the data. Anita C. Risch, Martin Schütz, Jennifer Firn and Uffe N. Nielsen were in charge of investigation and supervision. Anita C. Risch, Martin Schütz, Jennifer Firn and Uffe N. Nielsen arranged the logistics of the project. Marco Fioratti Junod, Anita C. Risch and Irene Cordero wrote the original draft. Anita C. Risch, Martin Schütz, Jennifer Firn, Irene Cordero, Nadia Chinn, Gabrielle Lebbink, Julia Holmes, Marcus Klein, Stephan Zimmermann and Uffe N. Nielsen contributed to reviewing and editing. Anita C. Risch, Jennifer Firn and Martin Schütz were in charge of project administration. All authors gave their final approval for publication.

## CONFLICT OF INTEREST STATEMENT

The authors do not have any conflict of interest to declare.

## STATEMENT OF INCLUSION

This study was conducted through collaboration between authors based in Switzerland and Australia, with data collection carried out in Australia. We acknowledge the importance of diverse perspectives in scientific research and have made every effort to incorporate relevant literature from the study area. Our approach aimed to ensure that local and regional contexts are well represented, contributing to a more inclusive and comprehensive understanding of the research topic.

## Supporting information


**Figure S1.** Time progression of temperature (a) and moisture values (b) in the soil profile. Data refer to the average (b and central panels of a), or maximum and minimum values in each 24‐h interval. The discontinuity points were used as knots for a splined linear model.
**Figure S2.** Relative abundance of bacteria phyla in the different herbivore exclusion treatments in both the native and invaded plots.
**Figure S3.** 16S inferred Faith's functional diversity of PICRUSt2 enzyme profiles in the herbivore exclusion treatments of both native and invaded plots.
**Figure S4.** (a) Relative abundance of fungal phyla. (b) Trophic guild profiles derived with FUNGuild in the different herbivore exclusion treatments in both the native and invaded plots.
**Figure S5.** Order level breakdown of nematode orders in the different herbivore exclusion treatments in both the native and invaded plots.
**Figure S6.** Cumulative abundance of recovered pitfall trap catches in the different herbivore exclusion treatments in both the native and invaded plots.
**Table S1.** Theoretical background and literature on which we built the a‐priori paths identified for the structural equation model.
**Table S2.** Modelling outcomes and estimate marginal means for selected parameters (separate file).
**Table S3.** Pairwise comparison from modelling the biomass from Kangaroo grass and other grasses in climate control cages and ∅ treatment cages. Synthesised from Chinn et al. (in review).

## Data Availability

All data supporting this article are available on the Envidat portal: https://www.doi.org/10.16904/envidat.677 [Fioratti et al. 2025] .
