# Supplementary material for: Herbivory mediates the response of below‐ground food webs to invasive grasses
Source: J Anim Ecol. 2025 Aug 29;94(10):2103–17. doi: 10.1111/1365-2656.70113 (PMC12484429; doi:10.1111/1365-2656.70113)
Supplement: Supplementary file 1 — Figure S1. Time progression of temperature (a) and moisture values (b) in the soil profile. Data refer to the average (b and central panels of a), or maximum and minimum values in each 24‐h interval. The discontinuity points were used as knots for a splined linear model. Figure S2. Relative abundance of bacteria phyla in the different herbivore exclusion treatments in both the native and invaded plots. Figure S3. 16S inferred Faith's functional diversity of PICRUSt2 enzyme profiles in the herbivore exclusion treatments of both native and invaded plots. Figure S4. (a) Relative abundance of fungal phyla. (b) Trophic guild profiles derived with FUNGuild in the different herbivore exclusion treatments in both the native and invaded plots. Figure S5. Order level breakdown of nematode orders in the different herbivore exclusion treatments in both the native and invaded plots. Figure S6. Cumulative abundance of recovered pitfall trap catches in the different herbivore exclusion treatments in both the native and invaded plots. Table S1. Theoretical background and literature on which we built the a‐priori paths identified for the structural equation model. Table S2. Modelling outcomes and estimate marginal means for selected parameters (separate file). Table S3. Pairwise comparison from modelling the biomass from Kangaroo grass and other grasses in climate control cages and ∅ treatment cages. Synthesised from Chinn et al. (in review). [file JANE-94-2103-s001.docx]

**
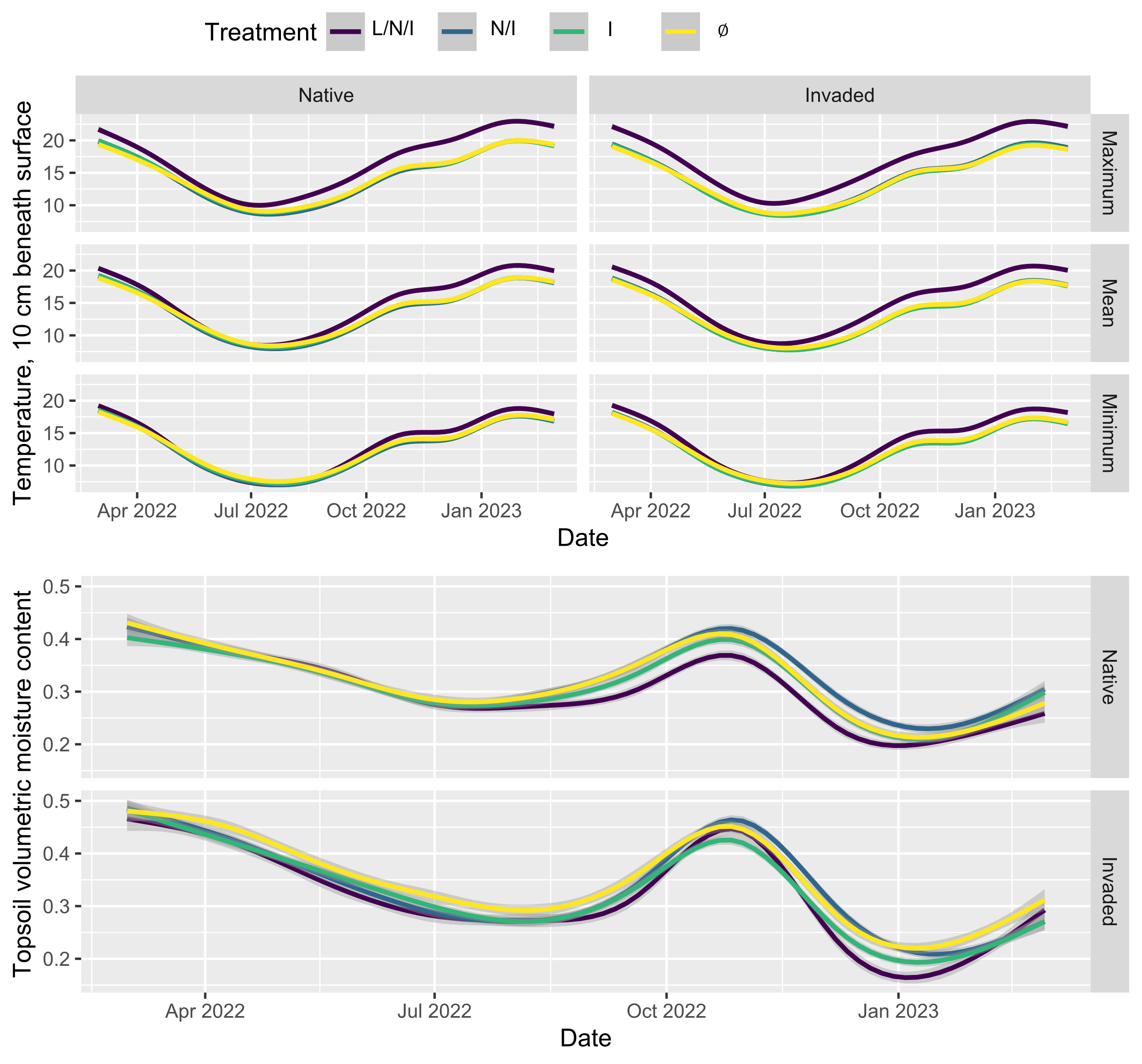
Supplementary materials**

Figure S1: Time progression of temperature (a) and moisture values (b) in the soil profile. Data refer to the average (b and central panels of a), or maximum and minimum values in each 24-hour interval. The discontinuity points were used as knots for a splined linear model.


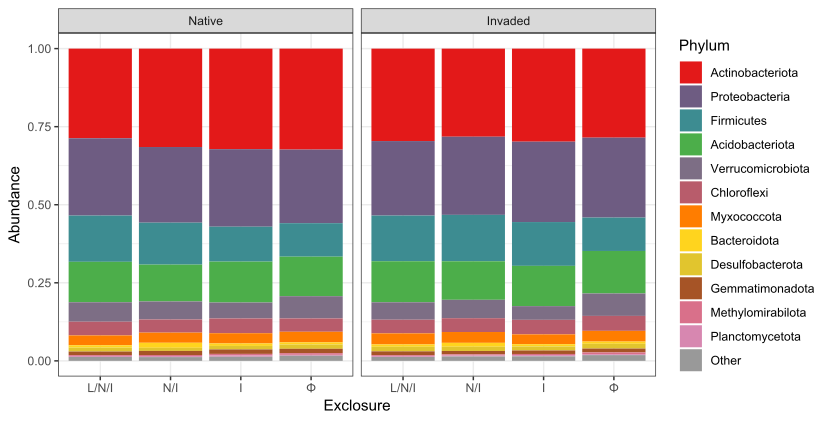


Figure S2: Relative abundance of bacteria phyla in the different herbivore exclusion treatments in both the native and invaded plots.


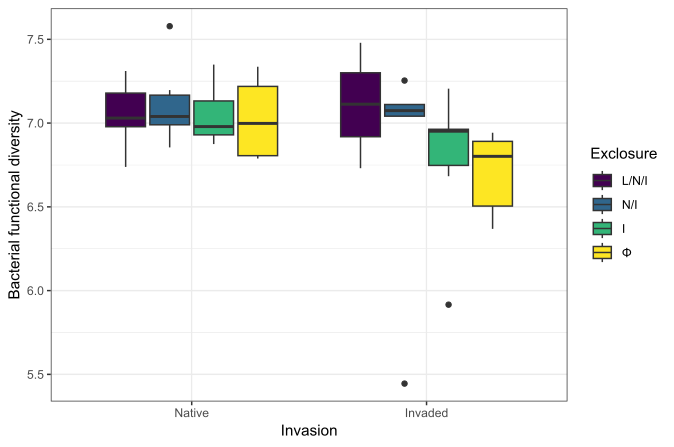


Figure S3: 16S inferred Faith’s functional diversity of PICRUSt2 enzyme profiles in the herbivore exclusion treatments of both native and invaded plots.

.


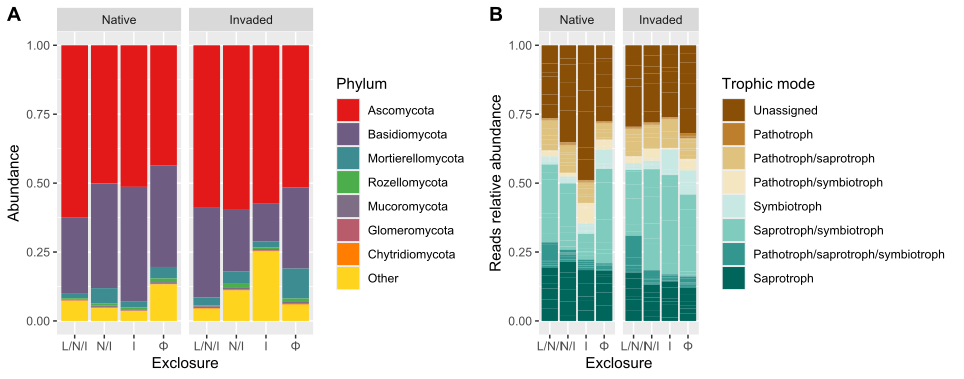


Figure S4 a) relative abundance of fungal phyla. b) trophic guild profiles derived with FUNGuild in the different herbivore exclusion treatments in both the native and invaded plots


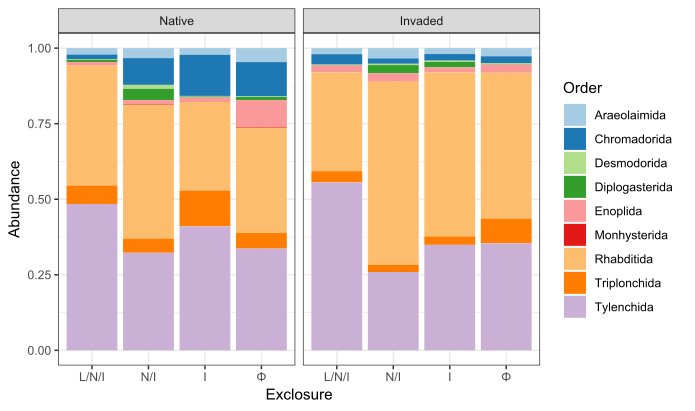


Figure S5 Order level breakdown of nematode orders in the different herbivore exclusion treatments in both the native and invaded plots


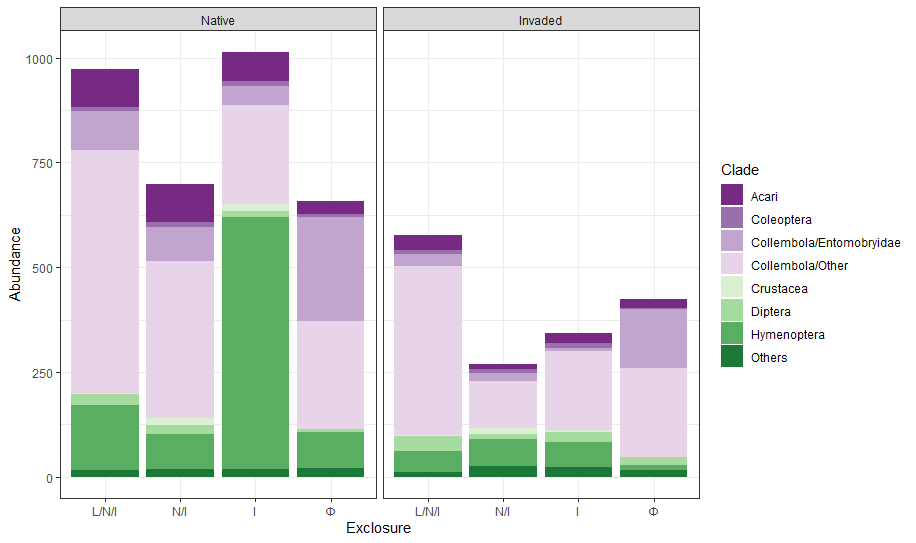


Figure S6 Cumulative abundance of recovered pitfall trap catches in the different herbivore exclusion treatments in both the native and invaded plots

Table S1: Theoretical background and literature on which we built the a-priori paths identified for the structural equation model.

| **Upstream** | **Downstream** | **References** |
| --- | --- | --- |
| Invasion | Litter | Csurhes et al., 2016 |
| Herbivory exclusion | Litter | Su et al., 2022 |
| Invasion | Soil temperature | Gibbons et al., 2017 |
| Herbivory exclusion | Soil temperature | Yan et al., 2018 |
| Invasion | Soil moisture | Wolkovich et al., 2009 |
| Soil temperature | Soil moisture | Yan et al., 2018 |
| Moisture | Saprotrophs | Rousk & Bååth, 2011 |
| Invasion | Saprotrophs | Zhang et al., 2019 |
| Invasion | Gallate pathway | Van Der Laan et al., 2008 |
| Bacterial diversity | Gallate pathway | Schmidt et al., 2013 |
| Saprotrophs | Phenol oxidase | Sinsabaugh, 2010 |
| Invasion | Phenol oxidase | Schmidt et al., 2013 |
| Soil temperature | Springtails | Briones et al., 2009 |
| Bacterial diversity | Springtails | Wood et al., 2017 |
| Litter | Springtails | Sauvadet et al., 2017 |
| Springtails | Mites | Ferguson & Joly, 2002 |
| Litter | Mites | Sauvadet et al., 2016 |
| Invasion | Nematodes | Zhang et al., 2019 |
| Litter | Nematodes | Sauvadet et al., 2016 |

Briones, M. J. I., Ostle, N. J., McNamara, N. P., & Poskitt, J. (2009). Functional shifts of grassland soil communities in response to soil warming. Soil Biology and Biochemistry, 41(2), 315–322. https://doi.org/10.1016/j.soilbio.2008.11.003

Csurhes, S., Leigh, C., & Walton., C. (2016). Weed risk assessment: African lovegrass Eragrostis curvula. Department of Agriculture and Fisheries; Biosecurity Queensland, July, 1–4. http://www.dpi.nsw.gov.au/__data/assets/pdf_file/0019/320158/African-lovegrass-management-web.pdf

Ferguson, S. H., & Joly, D. O. (2002). Dynamics of springtail and mite populations: The role of density dependence, predation, and weather. Ecological Entomology, 27(5), 565–573. https://doi.org/10.1046/j.1365-2311.2002.00441.x

Gibbons, S. M., Lekberg, Y., Mummey, D. L., Sangwan, N., Ramsey, P. W., & Gilbert, J. A. (2017). Invasive Plants Rapidly Reshape Soil Properties in a Grassland Ecosystem. MSystems, 2(2), 1–13. https://doi.org/10.1128/msystems.00178-16

Rousk, J., & Bååth, E. (2011). Growth of saprotrophic fungi and bacteria in soil. FEMS Microbiology Ecology, 78(1), 17–30. https://doi.org/10.1111/j.1574-6941.2011.01106.x

Sauvadet, M., Chauvat, M., Brunet, N., & Bertrand, I. (2017). Can changes in litter quality drive soil fauna structure and functions? Soil Biology and Biochemistry, 107, 94–103. https://doi.org/10.1016/j.soilbio.2016.12.018

Sauvadet, M., Chauvat, M., Fanin, N., Coulibaly, S., & Bertrand, I. (2016). Comparing the effects of litter quantity and quality on soil biota structure and functioning: Application to a cultivated soil in Northern France. Applied Soil Ecology, 107, 261–271. https://doi.org/10.1016/j.apsoil.2016.06.010

Schmidt, M. A., Kreinberg, A. J., Gonzalez, J. M., Halvorson, J. J., French, E., Bollmann, A., & Hagerman, A. E. (2013). Soil microbial communities respond differently to three chemically defined polyphenols. Plant Physiology and Biochemistry, 72, 190–197. https://doi.org/10.1016/j.plaphy.2013.03.003

Sinsabaugh, R. L. (2010). Phenol oxidase, peroxidase and organic matter dynamics of soil. Soil Biology and Biochemistry, 42(3), 391–404. https://doi.org/10.1016/j.soilbio.2009.10.014

Su, Y., Dong, K., Wang, C., & Liu, X. (2022). Grazing promoted plant litter decomposition and nutrient release: A meta-analysis. Agriculture, Ecosystems and Environment, 337(March), 108051. https://doi.org/10.1016/j.agee.2022.108051

Van Der Laan, M., Reinhardt, C. F., Belz, R. G., Truter, W. F., Foxcroft, L. C., & Hurle, K. (2008). Interference potential of the perennial grasses Eragrostis curvula, Panicum maximum and Digitaria eriantha with Parthenium hysterophorus. Tropical Grasslands, 42(2), 88–95.

Wolkovich, E. M., Bolger, D. T., & Cottingham, K. L. (2009). Invasive grass litter facilitates native shrubs through abiotic effects. Journal of Vegetation Science, 20(6), 1121–1132. https://doi.org/10.1111/j.1654-1103.2009.01110.x

Wood, J. R., Holdaway, R. J., Orwin, K. H., Morse, C., Bonner, K. I., Davis, C., Bolstridge, N., & Dickie, I. A. (2017). No single driver of biodiversity: Divergent responses of multiple taxa across land use types. Ecosphere, 8(11). https://doi.org/10.1002/ecs2.1997

Yan, Y., Yan, R., Chen, J., Xin, X., Eldridge, D. J., Shao, C., Wang, X., Lv, S., Jin, D., Chen, J., Guo, Z., Chen, B., & Xu, L. (2018). Grazing modulates soil temperature and moisture in a Eurasian steppe. Agricultural and Forest Meteorology, 262(12), 157–165. https://doi.org/10.1016/j.agrformet.2018.07.011

Zhang, P., Li, B., Wu, J., & Hu, S. (2019). Invasive plants differentially affect soil biota through litter and rhizosphere pathways: a meta-analysis. Ecology Letters, 22(1), 200–210. <https://doi.org/10.1111/ele.13181>

Table S2:Modelling outcomes and estimate marginal means for selected parameters (Separate file)

Table S3:Pairwise comparison from modelling the biomass from Kangaroo grass and other grasses in climate control cages and ∅ treatment cages. Synthesised from Chinn et al. (in review)

| **Biomass Type** | **Contrast** | **Odds ratio** | **SE** | **df** | **null** | **z-ratio** | **P-val** |
| --- | --- | --- | --- | --- | --- | --- | --- |
| **KG** | ∅/Control cage | 2.790493 | 1.33 | Inf | 1 | 2.1478437 | 0.08045375 |
| **OG** | ∅/Control cage | 0.6343355 | 0.2945302 | Inf | 1 | -0.9803241 | 0.589321507 |
